# Observations on Some Peculiar Varieties of “After Pains”

**Published:** 1852-08

**Authors:** C. Joel Hendrick

**Affiliations:** Auburn, Ind.


ART. III.—*Observations on some Peculiar Varieties of "After Pains."* By C. Joel Hendrick, of Auburn, Ind.

It is well known to physicians, that, after parturition, females are subject to periodical pains, which resemble, in some respects, the pains of labor, and are induced by the further contraction of the uterus after its contents have been expelled. These pains sometimes continue several days, and are very distressing to the patients, insomuch that it frequently becomes necessary to subdue or palliate them by the administration of medicine. Generally, opium or some of its preparations in full doses will arrest them promptly; but, from my own experience and observation, I am induced to believe that opium is too frequently given in cases where periodical pains occur soon after parturition, without a due consideration of the *character* of the pains. To confirm this opinion I will cite the following cases:—

Case 1.—Mrs. Brown, a very robust and healthy woman, was delivered of her first child in the spring of 1848. Nothing worthy of note occurred during the labor, and when I left the house, which was soon after her delivery, she appeared entirely comfortable. About four hours afterwards, however, I was again summoned to see her, and found her laboring under very severe pain, which recurred at regular intervals like ordinary after pains. As there was also occasional syncope with a frequent and feeble pulse, I was induced to examine the condition of the uterus, per vaginum. On attempting to examine the uterus, I was surprised to find the vagina much distended by an immense and very firm coagulum, which I immediately broke up and extracted. No hæmorrhage followed the extraction of the coagulum, and the pains immediately ceased.

CASE 2.—I was summoned in haste on the morning of the 4th January, 1850, to see Mrs. Williams who was reported to be in labor, under the care of Dr. E——. I arrived at the house about two o'clock, A. M., and found the woman suffering from very extreme pain, though she had been delivered, I was informed, about an hour before my arrival. The pains were decidedly periodic, though not at any time entirely absent, and where described by the patient as much worse than the pains she had suffered during labor. Dr. E—— informed me that the after pains, as he regarded them, had set in soon after the delivery of the placenta, and as they were unusually severe he had given a very large dose of morphine, which, however, had produced little or no impression upon them. I suggested the propriety of examining the condition of the uterus, and was requested by Dr. E—— to make the necessary examination. On introducing my hand I found protruding through the os uteri, to the extent of an inch or more, a substance which was highly sensitive to the touch, and which I concluded was the fundus of that organ. I therefore made firm pressure upon it, in consequence of which it suddenly receded within the os, upon which the pains *immediately* and *entirely* subsided.

In both the foregoing cases it is obvious that opium, to whatever extent its administration might have been carried, could not have afforded any material relief.

